# Criteria for the prescription of oral bisphosphonates for the treatment of osteoporosis in a series of women referred for tooth extraction

**DOI:** 10.4317/medoral.17681

**Published:** 2012-02-09

**Authors:** Márcio Diniz-Freitas, Javier Fernández-Feijoo, Paula Fernández-Montenegro, Antonio González-Mosquera, Emma Vázquez-García, Pedro Diz-Dios

**Affiliations:** 1 Oral Medicine and Oral Surgery Unit, Faculty of Medicine and Dentistry, University of Santiago de Compostela, Santiago de Compostela, Spain; 2 Special Needs Unit, Faculty of Medicine and Dentistry, University of Santiago de Compostela, Santiago de Compostela, Spain; 3 Primary Care Buccodental Health Unit, Lérez Health Centre, Pontevedra, Spain. Galician Health Department (SERGAS)

## Abstract

Objective: To evaluate the criteria for the prescription of oral bisphosphonates (OB) in a series of women with osteoporosis referred for tooth extraction. Study design: The study included 38 postmenopausal women on treatment with OBs. The following variables were analysed: age, weight, height, type of OB and duration of treatment, bone densitometry and risk factors for osteoporosis. In addition, the osteoporosis self-assessment tool (OST) was administered and collagen type I C-telopeptide (CTX) levels were measured. Results: Bone densitometry had only been performed in six patients (15.7%) before starting OB treatment. Based on the results of the OST, nine (23.6%) of the participants presented a low risk of osteoporosis. CTX levels were measured in 23 patients: 11 (47.8%) presented values below 150 pg/ml. Conclusion: Although all patients in the present series were on treatment with OBs, a large percentage did not satisfy the criteria for the initiation of treatment for postmenopausal osteoporosis.

** Key words:**Osteoporosis, oral bisphosphonates, osteonecrosis of the jaws.

## Introduction

Osteoporosis is defined as a skeletal disorder characterised by a reduction of the bone mass and a structural deterioration of bone tissue, leading to increased bone fragility. It mainly affects postmenopausal women and is associated with significant morbidity due to an increased risk of fractures (1). A number of pharmacological and non-pharmacological therapeutic initiatives have been proposed to prevent and treat postmenopausal osteoporosis. In Spain this has led to a steady increase in recent years in the prescription of drugs to combat the disease, particularly oral bisphosphonates (OB) (2). The efficacy of the OBs is related to their ability to inhibit bone resorption, the clinical correlate of which is a significant reduction in the prevalence of vertebral and non-vertebral fractures in postmenopausal women with osteoporosis (3).

New adverse effects of the prolonged administration of bisphosphonates have been detected in recent years; one of these, particularly important due to its associated morbidity, is osteonecrosis of the jaws (ONJ). Bisphosphonate-related ONJ is defined as an area of bone exposure in the maxillofacial region that persists for more than eight weeks in patients treated with bisphosphonates (at the time of the lesion or previously) and who have not received radiotherapy to that anatomical region. The management of established lesions of ONJ is complex and may require disfiguring surgical treatments with major functional and cosmetic sequelae. Although the relationship between treatment with bisphosphonates and ONJ is based on solid epidemiological evidence, osteonecrosis attributable to the use of OBs has been questioned more than that associated with the intravenous administration of these drugs (4). However, in a recently published retrospective multicentre study it was suggested that the relative frequency of ONJ in patients with osteoporosis treated with OBs is higher than previously estimated (5). Thus, if the prescription of OBs to some patients were not correctly indicated, they would be unnecessarily exposed to an increased risk of ONJ.

As tooth extractions are considered to be the principal trigger of OB-related ONJ (6), the present study was designed to evaluate the criteria applied for the prescription of OB in a series of postmenopausal women attending a dental clinic for tooth extractions.

## Material and Methods

The study group comprised 38 postmenopausal women who were on treatment with OBs for osteoporosis and who came to a primary care buccodental health unit of the Galicia Health Service (SERGAS) for tooth extractions during the period between June 2009 and June 2010. The mean age of the participants was 68.7 ± 7.6 years (range: 54-92 years). None of the women developed ONJ after performing the extraction (follow-up period: 3-12 months).

All the patients filled in a standardised health questionnaire to obtain the variables of interest, and the information was completed from a review of their medical histories in the computerised SERGAS data base. The following variables were analysed: age, weight, height, type of OB and duration of the treatment, bone densitometry study (dual x-ray absorptiometry [DXA]) and risk factors for osteoporosis ( Table 1). In addition, the osteoporosis self-assessment tool (OST) was administered; this estimates the risk of osteoporosis according to the weight and age of the patient. The cut-off point for the referral of patients to perform DXA is a score of 2. Prior to performing the extractions, the serum levels of collagen type I C-telopeptide (CTX) were determined (E 170 Elecsys, Roche Diagnostics, Mannheim, Germany).

**Table 1:**
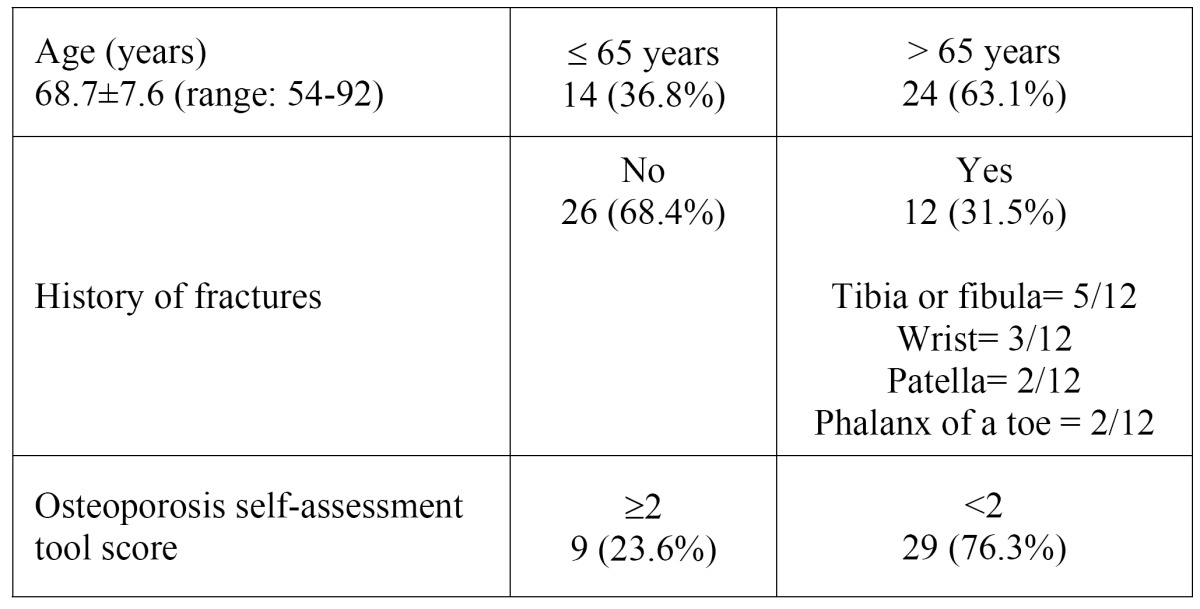
Risk factors for osteoporosis.

This project was approved by the Ethics Committee of the Galician Government and all participants signed a specific informed consent form before their inclusion in the study.

## Results

The most widely used OB in the study group was alendronate (50%), followed by risedronate (23.7%), ibandronate (23.7%) and the combination of alendronate with cholecalciferol (2.6%). The mean duration of treatment was 42.4 ± 40.4 months (range: 2-180 months); 16 patients (42.1%) had been on treatment for more than three years. The risk factors for osteoporosis and bisphosphonate-related ONJ are listed in  Table 1. Bone densitometry had only been performed in 6 patients (15.7%) before starting treatment and all of them had a T-Score (comparison of bone mineral density of the patient with that of a healthy individual of 30 years of the same sex and ethnic origin) less than -2.5 (2.5 standard deviations below the referent mean). A history of presumably osteoporotic fractures was detected in 12 patients (31.5%) ( Table 1). Twenty-nine participants (76.3%) were at risk of osteoporosis according to the results of the OST (OST score <2). CTX levels were measured in 23 patients and the mean value was 197.7 pg/ml; eight patients (34.8%) presented values below 100 pg/ml, three (13%) presented levels of 100-150 pg/ml and in the remaining 12 (52.2%) the values were over 150 pg/ml ( Table 2).

**Table 2:**
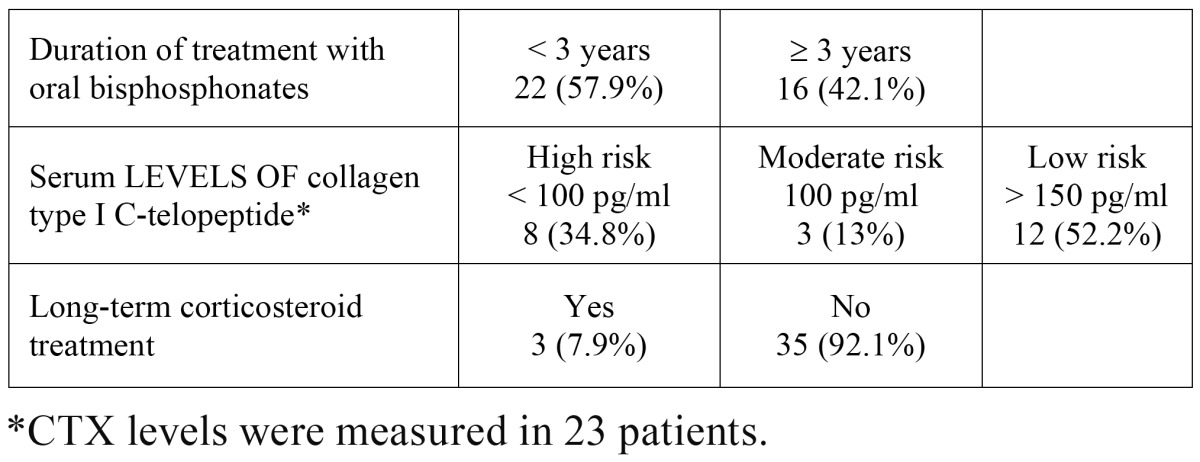
Risk factors for bisphosphonate-related osteonecrosis of the jaws in the present series.

## Discussion

In recent years there has been an alarming increase in the number of cases of ONJ in patients treated with OBs. Many of these patients present a poor oral health status (in the present series, 35.5% of the patients had calculus and the depth of periodontal probing was greater than 6 mm in 16.1%), meaning they have considerable requirements for dental treatment, including tooth extractions. This dental procedure is considered to be the most common triggering factor for OB-related ONJ (6). The management of established ONJ lesions is complex and the clinical course is difficult to predict; prevention and the control of risk factors is thus very important. One of the initial strategies to prevent ONJ associated with the administration of OBs to patients with osteoporosis would therefore be appropriate supervision of the prescription of these drugs.

Bone mineral density (BMD) is fundamental to the decision on whether or not to start pharmacological treatment of osteoporosis (7). DXA of the lumbar column and/or of the hip is the most reliable technique to determine the BMD (8) and the World Health Organization defines osteoporosis as a BMD T-score equal to or less than -2.5. Numerous guidelines, protocols and recommendations on strategies for the prevention and treatment of osteoporosis have been published, but the indication for densitometry continues to be a subject of controversy. In general, pharmacological treatment is administered to patients with a BMD T-Score equal to or less than -2.5, to those with a BMD T-score less than -2 in association with multiple risk factors, and to those with a history of osteoporotic fractures of the vertebral column or of the hip (9). Although a decrease in the BMD represents one of the most important risk factors for fractures due to osteoporosis, DXA had only been performed in 15% of patients in the present study. Another risk indicator commonly used to justify the initiation of pharmacological treatment for osteoporosis is a history of vertebral or hip fractures; this risk factor was not detected in any of the patients evaluated in the present study, although 31.5% of them presented fractures presumably of osteoporotic origin at other sites. In a recently published study performed by Felipe et al. (10) in a primary care centre in Parla, Madrid, Spain, it was found that only 51.8% of patients who were receiving treatment for osteoporosis satisfied the prescription criteria. On reviewing the medical histories, those authors found no record of a DXA scan in the majority of cases (73.1%) and that 39.5% of the patients had suffered at least one osteoporotic fracture. It was suggested that these results could be indicative of the difficulty of access to DXA in the Spanish national health system. As it is not feasible to determine the BMD in all at-risk patients in many countries in which access to DXA is not straightforward, a number of clinical-decision tools, principally in the form of simple questionnaires on the risk factors for osteoporosis, have been designed to identify those women with a low BMD who should be referred for DXA. In the present study we used the OST, as this is considered to be a useful index for identifying postmenopausal women with a low BMD (11). Based on the OST scores, 23.6% of the patients evaluated were classified as being at low risk and therefore referral for DXA would not be indicated.

The measurement of CTX levels has been recommended to establish the risk of bisphosphonate-related ONJ and to aid decision-taking in the dental setting, particularly with respect to surgical procedures (12). However, the usefulness of this parameter has subsequently been questioned and some authors do not consider it should be used routinely for this purpose (13,14). In a recently published study by Lazarovici et al. (15), it was demonstrated that there was a five-fold increase in the risk of developing bisphosphonate-related ONJ in subjects with CTX levels below 150 pg/ml compared to individuals with higher levels. Although 47.8% of the patients in the present study were identified as being at high risk according to the criteria proposed by Marx et al. (12), none developed ONJ after tooth extraction.

Osteoporosis is a major public health problem because it significantly increases the risk of fractures, particularly in untreated patients. In general, it is accepted that the benefits derived from treatment with OBs are greater than the relatively small risk of developing ONJ. Although all the patients in the present series were receiving OBs, the majority did not satisfy the criteria for the initiation of treatment for postmenopausal osteoporosis. Different protocols for the treatment of osteoporosis in postmenopausal women have been proposed aid decision-taking in the two most controversial aspects of osteoporosis: in which patients to perform DXA and in which to start pharmacological treatment (1,9). Adherence to standardised guidelines for the prescription of OBs in postmenopausal women could therefore help to improve the management of osteoporosis and reduce the prevalence of ONJ secondary to dental procedures.
